# Toluidine blue 1% eye drop versus optical coherence tomography for margin delimitation of ocular surface squamous neoplasia*

**DOI:** 10.1177/11206721231204188

**Published:** 2023-09-24

**Authors:** Flávia Benchimol Ferraz, Ana Marisa Pires Castelo Branco, Luiz Guilherme Ito da Cruz, Bruno F. Fernandes, Melina Correia Morales, Rubens Belfort Neto, Arthur Gustavo Fernandes

**Affiliations:** 1 Department of Visual Sciences and Ophthalmology, Federal University of São Paulo, São Paulo, SP, Brazil; 2 Department of Ophthalmology, Argumento Institute, Boucherville, QC, Canada; 3 Department of Anthropology and Archaeology, 2129 University of Calgary, Calgary, AB, Canada

**Keywords:** conjunctiva, diagnostic tests/Investigation, imaging, oncology

## Abstract

**Purpose:**

To compare the use of toluidine blue 1% eye drops with anterior segment optical coherence tomography (OCT) for the determination of tumour margins in patients with ocular surface squamous neoplasia (OSSN).

**Methods:**

The study was conducted from July 2020 to June 2021 at the Ocular Oncology department at the Federal University of São Paulo, Brazil. Slit-lamp photographs after toluidine blue staining and OCT of the anterior segment were taken on the same day from patients with OSSN. Photographs and OCT images were analyzed quantitatively using the software ImageJ and IMAGEnet®, respectively. The agreement between techniques was evaluated qualitatively through the Bland-Altman graph and quantitatively through intraclass correlation (ICC).

**Results:**

A total of 21 participants (71.43% males) with a clinical diagnosis of OSSN were included in the study. The average + SD diameter along the chosen axes was 4.43 ± 2.08 mm with OCT of 4.37 ± 2.03 mm with toluidine blue, a difference not statistically significant (p = 0.2891). The Bland-Altman analysis indicated a good qualitative agreement between the methods, with all cases inserted within the limits of agreement from −0.3217 to 0.4268. The ICC quantitative analysis showed an almost perfect agreement of 99.57% (95%CI: 98.96–99.83%; p < 0.001).

**Conclusions:**

Our findings showed that OCT and toluidine eye drops are equivalent in determining margins for tumour measurements, which is particularly relevant in low-income settings where anterior segment OCT is not available. The use of toluidine blue 1% could be an useful alternative to quantify the size of the tumour, help to monitor tumour growth, and outline margins for surgical planning.

## Introduction

Ocular Surface Squamous Neoplasia (OSSN) is the most common non-melanocytic tumour of the ocular surface, including a range of neoplastic conditions of the cornea and conjunctiva, from intraepithelial dysplasia to invasive squamous cell carcinoma. Risk factors include light pigmented skin, ultraviolet exposure, immunosuppression, human immunodeficiency virus (HIV), smoking, xeroderma pigmentosum, and human papillomavirus (HPV) infection.^1,2^ The incidence of OSSN has been reported as 0.13 to 1.9 per 100 000 persons, with a higher incidence in equatorial regions and older white males.^3^ The lesions are clinically diagnosed as elevated, gelatinous, papilliform, or leukoplakic limbal lesions that can be moved freely over the sclera and have characteristic tufts of blood vessels within. Involvement of the cornea is common, usually in the form of a grey, opaque, and thickened epithelium. The diagnosis is typically made on a clinical basis and can be confirmed by impression cytology or biopsy followed by histopathologic examination.^4^ Treatment options for OSSN include surgery, perilesional injections, and topical treatment with chemotherapeutic drops.

Topical treatment using mitomycin C, 5-fluorouracil, and interferon have shown effectiveness as a non-invasive method for treating OSSN in specific cases.^5^ Traditional surgical therapy for OSSN consists of wide excision using a no-touch technique,^6^ with double cryotherapy to the surgical margins and pathologic examination of the sample, including the margins of the specimen.^7^ During surgery, the lesion is removed with a safety margin of 3 to 4 mm to ensure the removal of the entire tumour, which illustrates the importance of having a method that accurately defines tumour margins.

While originally pioneered for the posterior segment, the Optical Coherence Tomography (OCT) also allows in vivo imaging of the anterior segment, which is particularly helpful in managing ocular surface tumours, corneal pathologies, refractive surgery planning, and intraoperative uses, such as in lamellar surgeries.^1^ OCT can be a non-invasive tool for diagnosing epithelial neoplasms and is becoming a critical element in the management of OSSN.^1,5,8,9^ OCT images can help identify the true extent of OSSN lesions beyond what is apparent with the slit lamp examination, providing proper guidance for margins delimitations in surgical planning.^5^

Staining ocular surface lesions with rose bengal, lissamine green, and toluidine blue have been described as tools to assist in delineating the margins of ocular surface tumours. Toluidine blue is an acidophilic metachromatic dye that penetrates the nuclei of cancer cells, where there is a selective affinity for nucleic acids. It also accumulates in the intercellular space, staining abnormal tissues in a dark blue colour.^10^ Malignant tissues stain more often than healthy epithelia due to their abundance of mitotic nuclear material and low cell-to-cell adhesion.^10^ The observation of toluidine blue staining intensities and patterns can help in diagnosing OSSN. Dark blue and/or mixed intensity and a stippled pattern of TB staining have high sensitivity (86%) and specificity (94.74%) for the diagnosis of these lesions.^11^ The high negative predictive value suggests OSSN is relatively unlikely if staining is negative.^10^

Considering the importance of a precise delimitation of tumour margins and the challenging access to OCT exams in low-income regions, the purpose of the current study was to compare the tumour dimensions measured by OCT and by 1% toluidine blue staining. Toluidine blue eye drops are more accessible than OCT and can be an alternative for margins delimitation and determining the extent of a lesion in patients with OSSN. To the best of our knowledge, this is the first study comparing the margins specified by anterior segment photography with toluidine blue and anterior segment OCT.

## Methods

The study was conducted from July 2020 to June 2021 at the Ocular Oncology department at the Federal University of São Paulo (UNIFESP), São Paulo, Brazil. It was carried out in accordance with the tenets of the Declaration of Helsinki after approval by the Research Ethics Committee of UNIFESP. Informed consent was obtained from all participants. Patients with a clinical diagnosis of OSSN in the bulbar conjunctiva or cornea up to a length of 12 millimetres were recruited. Cases were selected in which TB eye drops well impregnated the whole lesion in dark blue and/or mixed intensity with a stippled pattern of TB staining, showing good delimitation. Cases that impregnated with light blue were excluded. Cases of OSSN in the tarsal conjunctiva, caruncle, or fornix, and lesions that occupied more than one quadrant were excluded because of the challenge of measuring them accurately. Hyperpigmented lesions of the ocular surface or participants that had received previous ocular surgeries and had a past or current use of topical chemotherapy were also excluded.

Lesions were photographed using a DC-3 digital camera (Topcon®, Tokyo, Japan), attached to the slit lamp, with 10x magnification focusing on the lesion, exposing all the borders, after instillation of 1 drop of anesthetic eye drops (proxymetacaine cloridrate 5 mg/ml; Alcon Laboratórios do Brasil, SP, Brazil) and one drop of toluidine blue 10 mg/ml (Eye Pharma, SP, Brazil). Slit lamp photos were taken 1 min after installation of TB eye drops in all patients in the study. The anterior segment OCT was performed on the same day using the commercially available Triton SS-OCT (Topcon, Tokyo, Japan), which has a maximum scan length of 16 millimetres. The OCT protocol included 12 circumferential scans in each clock hour around the tumour and horizontal scans covering the whole lesion plus and an additional 4 mm margin. OCT images were measured using the equipment's software IMAGEnet. Images obtained at the slit lamp were measured using the ImageJ application (developed by Wayne Rasband at the National Institute of Mental Health, USA, in Java language), adjusting the scale according to the manufacturer's instructions. A first ophthalmologist was responsible for determining at random the axis used for comparing both methods in each patient. Two other ophthalmologists with extensive experience in ocular oncology independently performed the measurements using the slit lamp photographs and OCT images based on the axes previously chosen by the first ophthalmologist. The grading ophthalmologists determined the tumour margins of the lesion along the selected axis according to the clinical features found on the OCT images and the toluidine staining on slit-lamp photographs.

Data were analyzed using the STATA 14.0 software (StataCorp LP, College Station, TX, USA). Frequency tables were used for descriptive analyses. Each parameter was considered as a mean from the measurements by graders 1 and 2. Wilcoxon test was used to compare the measurements from the two techniques. The Bland-Altmann and intraclass correlation (ICC) analysis were applied to investigate, respectively, the qualitative and quantitative agreement between the measurements. For all tests, a p-value < 0.05 was considered significant.

## Results

A total of 21 participants with a clinical diagnosis of OSSN were included in the study. Table 1 shows their clinical and demographic characteristics.

**Table 1.** Clinical and demographical characteristics of patients with ocular surface squamous neoplasia.

Sex; N (%)	
Male	15 (71.43%)
Female	6 (28.57%)
Age; mean ± SD (range)	60.08 ± 14.3
Flat/nodular lesion; N (%)	5 (19 to 93)
Flat	7 (33.33%)
Nodular	14 (66.67%)
Papillary lesion; N (%)	
Yes	5 (23.81%)
No	16 (76.19%)
Leukoplakic lesion; N(%)	
Yes	7 (33.33%)
No	14 (66.67%)
Location; N(%)	
Conjunctiva	16 (79.19%
Cornea/Conjunctiva	5 (23.81%)

**Figure 1.**
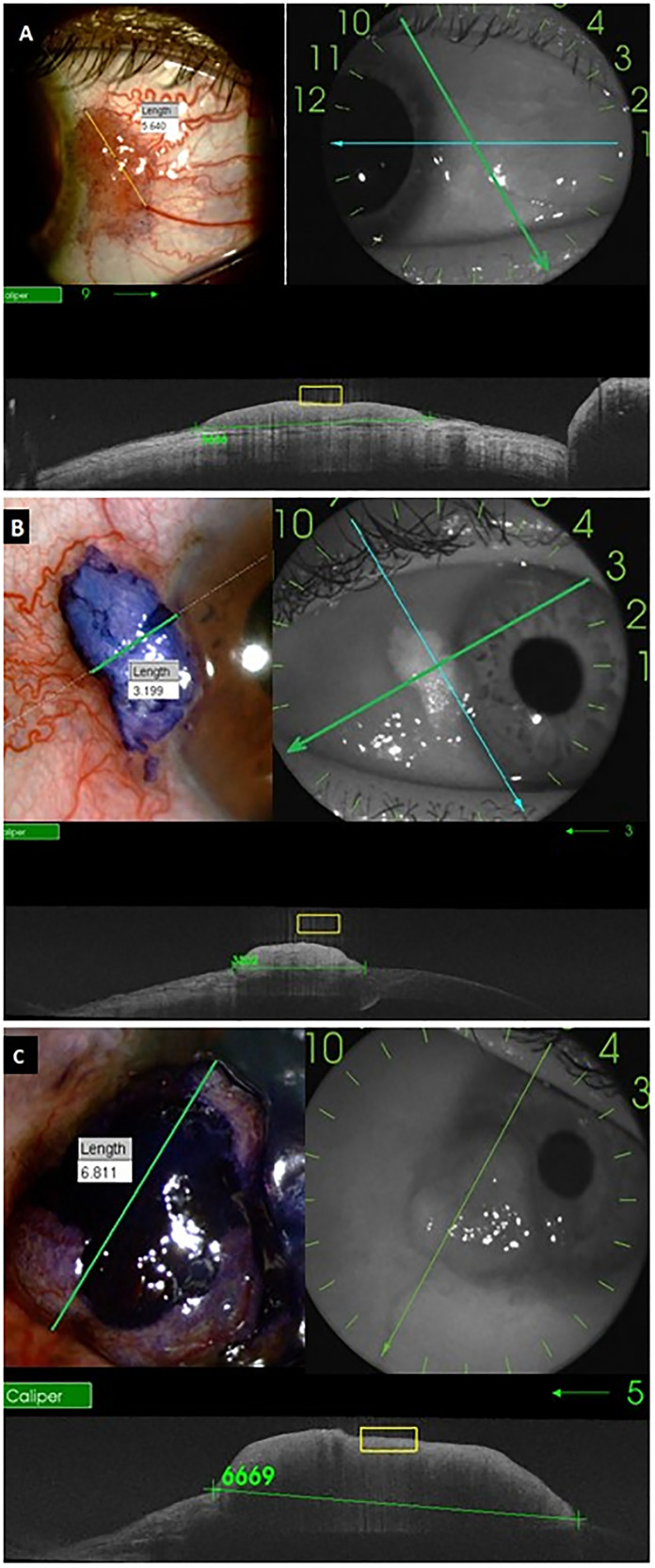
Comparison of measurements obtained from slit lamp photographs following toluidine blue staining and anterior segment OCT images. (A) 48 years old participant, nodular and papilliform lesion; (B) 65 years old participant, nodular and leukoplakic lesion; (C) 59 years old participant, nodular lesion.

**Figure 2.**
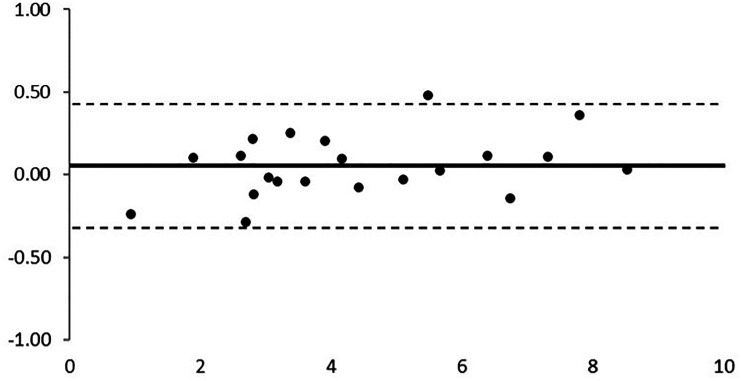
The Bland-Altman analysis indicated a good qualitative agreement between the slit lamp photographs following toluidine blue staining and anterior segment OCT images, with all cases within the limits of agreement from −0.3217 to 0.4268.

Figure 1 shows examples of OCT and slit-lamp images from different OSSN cases that the two grading ophthalmologists used to measure the lesions. All studied lesions showed positive staining with the TB dye. There were no pure corneal lesions without conjunctival involvement. The average + SD measurement of the diameter of the OSSN along the chosen axes with toluidine blue was 4.37 ± 2.03 mm (median 3.80 mm; range 1.05-8.50 mm), compared to 4.43 ± 2.08 (median 4.01 mm; range 0.81-8.54 mm) with OCT. Wilcoxon Test indicated no statistically significant difference between the measurements from the two techniques (p = 0.2891). The Bland-Altman analysis showed a good qualitative agreement between the methods, with all cases within the limits of agreement from −0.3217 to 0.4268 (Figure 2). The ICC quantitative analysis showed an almost perfect agreement of 99.57% (95%CI: 98.96–99.83%; p < 0.001).

## Discussion

With the move towards also less invasive topical therapies for OSSN, less invasive diagnostic options have also been proposed. Those include clinical examination by experience ocular oncologists, staining with toluidine blue and other dyes, impression cytology, in vivo confocal microscopy, and OCT.^5^ The current resolution of HR-OCT devices allows the identification of OSSN and its differentiation from other ocular surface lesions and presents an excellent correlation with histopathology.^1^ A pilot study with HR-OCT showed that it could successfully identify tumour margins coinciding with the histological margin of the tumour.^1^ Similar to histopathologic findings, HR-OCT images of OSSN show thickened, hyperreflective epithelium and an abrupt transition to the healthy epithelium. However, while HR-OCT has excellent specificity and sensitivity for diagnosing OSSN, it can take multiple scans until the exact tumour margin is identified; and a trained professional is needed to perform the exam. While in most cases of OSSN tumour margins show the classic features on OCT images, in some cases, the non-neoplastic epithelium may be somewhat hyperreflective due to non-neoplastic tissue changes. In these cases, the margin is characterized by the transition from a thickened to a thin epithelium, without an abrupt change in reflectivity.

Although histopathology is the gold standard for assessing deep margins in cases of OSSN, HR-OCT has excellent specificity and sensitivity for diagnosing OSSN, and has been able to identify lateral tumour margins, later confirmed by histopathology in all cases.^1^ Although HR-OCT has the potential to become a non-invasive gold standard for the identification of the lateral extent of OSSN lesions, a trained professional is needed to perform the exam, sometimes multiple scans are needed to accurately identify tumour margins, and it is expensive and difficult to access by low-income population or in developed countries with a unified public health system. Conversely, Toluidine blue can be ordered at specialty pharmacies at a cost of approximately USD 20 for a 5 ml bottle, equivalent to 100 drops. Since one drop is enough per exam, and a slit lamp is a ubiquitous tool in eye clinics, each test would cost only USD 0.2. Toluidine blue eye drop is already a known screening tool, given its high sensitivity and specificity for OSSN.^12^ The results from the present study showed that the determination of margins and tumour measurements in OSSN showed an excellent qualitative/quantitative agreement between anterior segment OCT images and slit lamp photographs following toluidine blue 1% staining.

One of the limitations to consider in the study is that the graders were ophthalmologists who specialized in Ocular Oncology and were familiar with OSSN. Graders that are less knowledgeable about the disease may not be as accurate in identifying the proper margins. Another limitation was that we excluded patients with a past or current history of topical chemotherapy, as those lesions tend to stain poorly with toluidine. Consequently, we cannot assert the usefulness of the method in those patients, and neither with OSSN in the tarsal conjunctiva, caruncle, or fornix that were also excluded due to limitations for OCT examination. Further studies, including non-specialized graders and a broader spectrum of OSSN, are warranted to expand the benefit of such tests to as many patients as possible.

Our findings showed no significant differences between OCT and toluidine blue 1% staining to determine OSSN lateral margins. Toluidine blue eye drops could become an easy-to-perform and inexpensive method to accurately monitor tumour progression and assist with surgical planning, especially in low-income settings where anterior segment OCT is unavailable.
